# Immunohistochemical Expression of Glucose Transporter-1 in Oral Epithelial Dysplasia and Different Grades of Oral Squamous Cell Carcinoma

**DOI:** 10.3390/medicina61040557

**Published:** 2025-03-21

**Authors:** Rahma Gamal Mostafa, Mohammad Ibrahim Hashim, Ahmed Abdulwahab Bawahab, Razan Abed A. Baloush, Mohammed S. Abdelwahed, Abdulkarim Hasan, Khadiga A. Ismail, Nageh Rady Abd-Elhameed, Ahmed Embaby, Abd El Rahman M. Sharfeldeen

**Affiliations:** 1 Department of Oral and Maxillofacial Pathology, Faculty of Dentistry, Assiut University, Assiut 71524, Egypt; 2 Department of Basic Medical Sciences, College of Medicine, University of Jeddah, Jeddah 23218, Saudi Arabia; 3 Department of Pathology, Faculty of Medicine, Al-Azhar University, Cairo 11561, Egypt; 4 Department of Clinical Laboratory Sciences, College of Applied Medical Sciences, Taif University, Taif 21944, Saudi Arabia; 5 Department of Pathology, Faculty of Medicine, Al-Azhar University, Assiut 71524, Egypt; 6 Department of Surgical Oncology, Faculty of Medicine, Al-Azhar University, Cairo 11561, Egypt; 7 Department of General Surgery, Lister Hospital, Stevenage SG1 4AB, UK; 8 Department of Oral Pathology, College of Dentistry, City University Ajman, Ajman P.O. Box 18484, United Arab Emirates

**Keywords:** oral epithelial dysplasia, glucose transporter-1, prognostic marker, oral squamous cell carcinoma

## Abstract

*Background and Objectives*: Glucose Transporter-1 (GLUT1) is the key target gene for hypoxia-inducible factor (HIF), which helps cells uptake glucose during cell division, malignant transformation, and nutrient depletion. Cancer hypoxia is a well-known condition caused by an oxygen imbalance in the cancer microenvironment. During chronic hypoxia, certain cancer cells can survive and adapt. These cellular alterations can make cancer more aggressive, causing invasion and metastasis. The study investigated the presence of GLUT1 in oral epithelial dysplasia (OED) and various histopathological grades of oral squamous cell carcinoma (OSCC) to assess the significance of GLUT1 as a prognostic indicator. *Material and Methods*: A total of 40 samples of tissue blocks, including 5 cases of normal oral mucosa, 5 cases of epithelial dysplasia, and 30 cases of OSCC with 10 cases each of well-differentiated, moderately differentiated, and poorly differentiated OSCCs, these cases were diagnosed using the Hematoxylin and Eosin (H&E) staining technique. GLUT1 expression was assessed using immunohistochemical staining, which involved evaluating the location of the stain and the percentage of staining. *Results*: The mean area percent was highest in poorly differentiated cases (47.37) and lowest in well-differentiated cases (13.42). In poorly differentiated cases, diffuse expression was observed in almost all malignant cells, exhibiting membrane, cytoplasmic and nuclear staining. A significant difference (*p* < 0.001) between all groups in regard to immunostaining was detected. *Conclusions:* GLUT1 expression increased from oral epithelial dysplasia to oral squamous cell carcinoma histological grades. GLUT1 in actively dividing cells may reflect the tumor’s aggressiveness and treatment response. Hypoxia increases this marker’s expression, indicating division and proliferation.

## 1. Introduction

Oral cancer is a widespread global health problem characterized by substantial rates of illness and death. Despite the existence of preventive strategies, this cancer continues to be highly prevalent, highlighting the necessity for enhanced diagnostic and prognostic techniques [1].

More than 90% of OSCCs are the most prevalent and deadly malignant tumors in the head and neck area. Approximately 380,000 new cases are anticipated annually, making it one of the most common tumors globally. This tumor accounts for 2% of all cancer cases and will rank eighteenth in 2020 [2].

Oral potentially malignant disorders (OPMDs) frequently occur before the development of neoplastic tumors [3]. These lesions, including leukoplakia, oral lichen planus, and oral lichenoid lesions, are part of this group [4]. Previously, conditions such as OED, proliferative verrucous leukoplakia, submucous fibrosis, and HPV-associated dysplasic lesions were categorized as OPMDs [5].

The majority of instances of OSCC are detected in advanced stages. That may be attributed to the patient’s lack of awareness, as most cases are asymptomatic in the early stages. There is a requirement to create novel immunohistochemistry markers for the early detection of OSCC patients to commence therapy and enhance the survival rate of this severe disease [6,7].

Glucose transporters are membrane proteins transporting hexoses like glucose and fructose through plasma membranes. Glucose Transporter-1 (GLUT1) is the main target gene for hypoxia-inducible factor (HIF), enabling glucose entry into cells during settings with heightened metabolic demands, such as cell division, malignant transformation, and nutrient insufficiency [8].

GLUT1 is a glycoprotein located in the cell membrane. Its primary function is to transfer glucose into cells without sodium ions. Research has shown changes in the expression of GLUT1 in several lesions that can potentially become cancerous or are already cancerous [9].

Cancer hypoxia is a widely recognized occurrence that arises from an inequity between the availability and utilization of oxygen within the microenvironment of the cancer. During prolonged periods of low oxygen levels, specific cancer cells can survive and undergo adaptations in response to hypoxia. These cellular changes can contribute to a more aggressive cancer phenotype, leading to invasion and metastasis [10].

GLUT1 may play an essential role in carcinogenesis. High GLUT1 expression has been reported in head and neck squamous cell carcinoma [11]. GLUT-1 expression in various head and neck tumors has shown different correlations with factors such as progression and prognosis [12]. This study investigated the presence of GLUT11 in oral epithelial dysplasia and various histological grades of OSCC to assess the significance of GLUT1 as a prognostic indicator.

## 2. Material and Methods

After the study received ethical approval from the Faculty of Dental Medicine ethics committee, Al-Azhar University in Assiut, Egypt, we collected forty formalin-fixed, paraffin-embedded tissue specimens from our institution’s pathology archives in Assiut, Egypt. Related clinical and demographic data were retrieved from medical files.

The specimens were initially preserved in a 10% buffered formalin solution for 24 h following the extraction of third molars. Group I comprised five normal oral mucosa (NOM) samples, serving as controls, which were collected from the gingival and vestibular mucosa. Group II included five cases, as defined by the OED. Group III consisted of thirty cases of OSCC, which were further classified into ten cases each of well-differentiated, moderately differentiated, and poorly differentiated OSCC.

The histopathological diagnosis and grading were reviewed by at least two pathologists (on-site or remote examination) to confirm the included cases. Exclusion criteria included non-Egyptian patients and cases with missing data or insufficient material in the cell block.

### 2.1. Immunohistochemical Study

Paraffin-embedded tissue specimens were cut into 4 μm thick sections and subjected to immunohistochemistry using the streptavidin biotin-peroxidase method. The primary antibody used was the rabbit monoclonal antibody GLUT1 EP141.

The sections were deparaffinized by immersion in xylene twice for five minutes each, followed by rehydration using a graded ethanol series (100%, 95%, and 70%). The slides were then washed in phosphate-buffered saline (PBS) to facilitate antigen recovery. Antigenic sites were revealed using heat-induced antigen retrieval with citrate buffer (pH 6.0) or Tris-EDTA buffer (pH 9.0), typically by heating at 95 °C for 15–20 min in a microwave or water bath. Following a 20 min cooling period at room temperature, the slides were washed with PBS to remove buffer residues.

To mitigate background staining, endogenous peroxidase activity was inhibited with a peroxidase-blocking solution for 10 min, followed by washing with PBS. A blocking buffer containing 5% bovine serum albumin (BSA) or normal serum was applied for 30 min to prevent nonspecific binding. The primary antibody, anti-GLUT1, was then applied at an optimum dilution (typically 1:100–1:500) and incubated overnight at 4 °C or for 1–2 h at room temperature. After incubation, the slides were washed three times with PBS, each lasting five minutes.

A secondary antibody conjugated to HRP or alkaline phosphatase, corresponding to the host species of the primary antibody, was applied and incubated for 30 min at room temperature. The slides were then washed three more times in PBS before the addition of a chromogenic substrate, such as 3,3′-diaminobenzidine (DAB) for HRP-based detection, producing a brown stain. The reaction was monitored under a microscope and terminated by washing the slides with distilled water.

For contrast, sections were counterstained with hematoxylin for 30 to 60 s, then rinsed in running tap water. The slides were subsequently dehydrated using a graded ethanol series, cleared with xylene, and mounted with a permanent medium for visualization. Under microscopic examination, positive GLUT1 expression appeared as brown membrane staining in cells, while negative regions remained unstained except for the counterstain. Internal positive controls, including red blood cells, exhibited GLUT1 positivity to ensure staining reproducibility. This refined procedure ensured precise and consistent detection of GLUT1 expression across different tissues, facilitating research and diagnostic applications, such as tumor characterization and metabolic investigations.

Microscopic images were captured using a SOPTOP EX20 biological microscope (Ningbo Sunny Instruments Co., China, Yuyao), an HD camera (model No. XCAM1080PHB) (manufactured by ToupTek Photonics, Hangzhou, China), and Image View software at ×100 and ×200 magnification.

Immunohistochemical stained sections were examined using high power fields (X200) by light microscope, and the positive reaction’s most homogenous areas were evaluated. The image analyzer computer system applying Imagej 1.53e software (USA) was used for automated measurement of area percent of Glut-1 positivity. It was performed in a standard frame area of 5.04 × 10^6^ µm^2^.

### 2.2. Statistical Methods

The results were evaluated through statistical analysis using the SPSS software (version 24). The Shapiro–Wilk test of normality was employed to assess the normality assumption for all continuous variables. The One-way analysis of variance (ANOVA) test was used to determine the presence of a statistically significant difference among the groups under investigation. Subsequently, the Tukey–Kramer Post hoc test was conducted to further examine the statistically significant findings. Statistical significance was determined by considering *p*-values less than or equal to 0.05.

## 3. Results

### 3.1. Immunohistochemical Findings of GLUT-1

**Figure 1:**
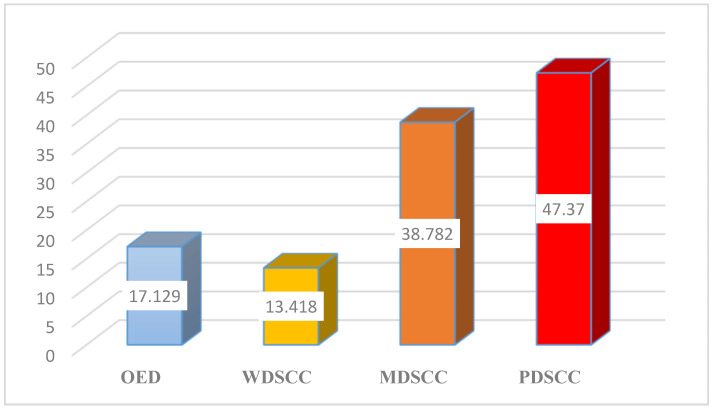
Mean area percentage of GLUT1 among groups.

The greatest mean area percent was recorded in poorly differentiated cases (47.37), whereas the lowest value was recorded in well-differentiated cases (13.42). One-way analysis of variance (ANOVA) test revealed that the difference between all groups was statistically significant (*p* < 0.001) with Cohen’s d effect size = 10.4 and 95% CI [7.3176, 13.4266]. Tukey’s post hoc showed that all groups were significantly different from each other (when comparing every two groups) (Table 1, Figure 1).

These results suggest a potential role of differentiation status in influencing the observed parameter, warranting further investigation into the underlying biological mechanisms.

Tukey’s post hoc test means sharing the same superscript letter is not significantly different.

### 3.2. Evaluation of GLUT-1 Immunostaining

**Figure 2:**
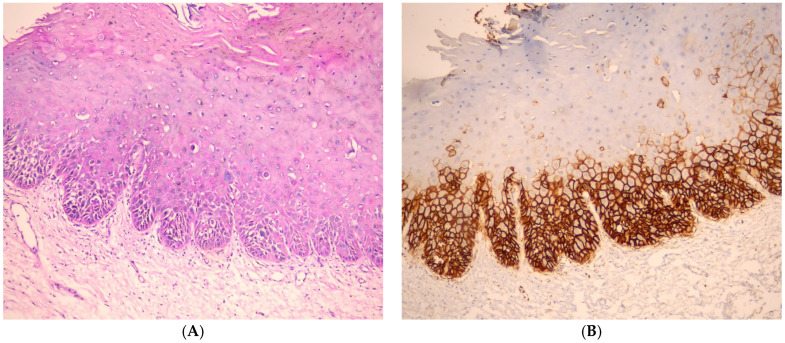
(**A**) Photomicrograph showing oral epithelial dysplasia (H&E, ×100). (**B**) Photomicrograph showing moderate expression of GLUT1, which extends to the mid-spinous layers in oral epithelial dysplasia (IHC, ×100).

In oral epithelial dysplasia cases, GLUT1 immunostaining was more intense in the basal and suprabasal layers of the epithelium, with a gradual reduction in reactivity observed in the most superficial layers. This staining pattern suggests an increased metabolic demand in proliferative regions, consistent with cellular adaptation to hypoxia. Notably, GLUT1 expression was predominantly localized at the tumor periphery, where cells are likely experiencing a higher proliferative rate, while significantly weaker staining was observed in the central regions of the tumor areas, possibly due to reduced metabolic activity or necrotic changes (Figure 2).

GLUT1-positive expression was identified in all cases of oral squamous cell carcinoma (OSCC), indicating its ubiquitous presence across different grades of malignancy. A detailed comparison of GLUT1 immunoexpression in relation to tumor grade revealed a progressive shift in staining distribution. As the grade of OSCC increased, the expression pattern changed from being predominantly localized in peripheral cells of tumor islands to a more diffuse pattern, encompassing both peripheral and central tumor cells. This shift may reflect altered tumor microenvironment conditions, such as increased glucose demand and hypoxia-driven metabolic adaptation in more aggressive tumors.

**Figure 3:**
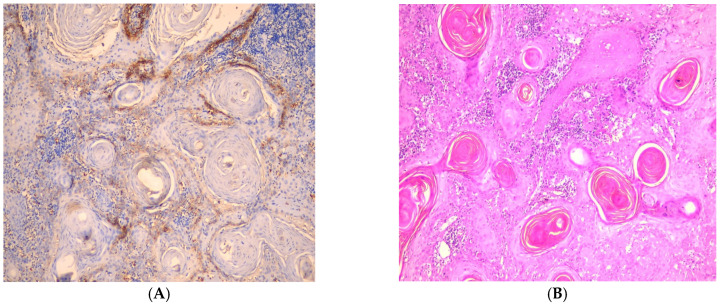
(**A**) Photomicrograph showing well-differentiated OSCC (H&E, ×100). (**B**) Photomicrograph showing mild expression of GLUT1 in well-differentiated OSCC showing peripheral tumor cells showing membranous expression of GLUT1 (IHC, ×100).

**Figure 4:**
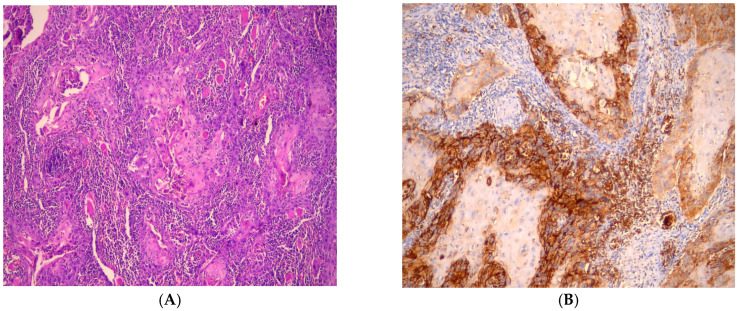
(**A**) Photomicrograph showing moderately differentiated OSCC (H&E, ×100) (**B**) Photomicrograph showing moderate expression of GLUT1 in tumor islands of moderately differentiated OSCC showing intense GLUT1 expression in peripheral cells of tumor island (IHC, ×100).

In well-differentiated OSCC cases, immunostaining was most pronounced in cells at the periphery of tumor cell nests, while keratin pearls exhibited negative expression. The observed membranous localization of GLUT1 suggests its functional role in glucose transport at the tumor-stroma interface, where active proliferation occurs (Figure 3). In moderately differentiated OSCC cases, the immunostaining pattern was more heterogeneous, with patchy expression detected in the central tumor cells, indicating a more diffuse metabolic adaptation. Interestingly, positive GLUT1 immunoreactivity was also noted in the endothelial lining of blood capillaries, suggesting a potential role in tumor-associated angiogenesis (Figure 4). These findings underscore the dynamic nature of GLUT1 expression in OSCC progression and its potential relevance as a biomarker for tumor metabolism and aggressiveness.

**Figure 5:**
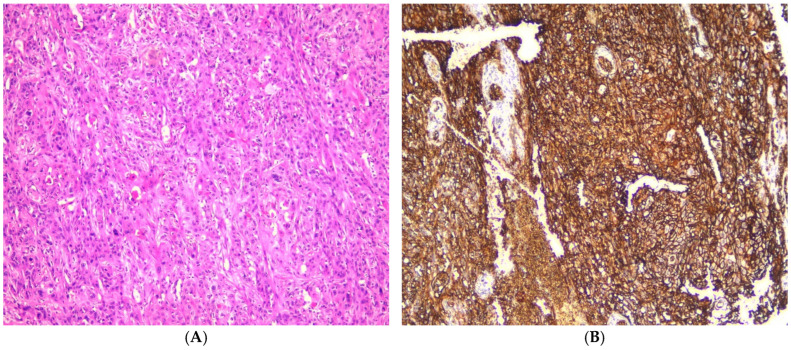
(**A**) Photomicrograph showing poorly differentiated OSCC (H&E, ×100). (**B**) Photomicrograph shows intense expression of GLUT1 in peripheral and central cells of tumor island in poorly differentiated OSCC with membranous and cytoplasmic staining (IHC, ×100).

In poorly differentiated cases, diffuse expression was observed in almost all malignant cells, exhibiting membrane, cytoplasmic and nuclear staining (Figure 5).

## 4. Discussion

Progress in surgical procedures, radiation, and chemotherapy has enhanced organ preservation and the overall quality of life while reducing morbidity. Nevertheless, further comprehension of the molecular pathways that govern the progression from normal epithelium to pre-malignancy to invasive oral cancer is imperative to enhance the long-term survival rates of affected patients [13].

Oral squamous cell carcinoma (OSCC) is a prevalent malignancy often linked to poor prognosis. OSCCs demonstrate the Warburg effect, relying heavily on an increased glucose supply to sustain tumor growth and metabolism. In various cancers, glucose transporters (GLUTs) are overexpressed, playing a crucial role in tumor progression. Their excessive production has been associated with treatment resistance, adverse clinical outcomes, and reduced overall survival rates [14].

Aerobic glycolysis, often referred to as the Warburg effect, produces significantly less adenosine triphosphate (ATP) per glucose molecule compared to mitochondrial oxidative phosphorylation (OXPHOS). However, the rate of glucose metabolism via aerobic glycolysis is markedly higher, allowing cells to rapidly generate lactate from glucose at a rate 10 to 100 times faster than the complete oxidation of glucose through OXPHOS [15]. This metabolic shift enables proliferating cells to sustain their energetic and biosynthetic demands despite their lower ATP.

For a cell—whether normal or altered—to actively proliferate, it must duplicate its DNA content along with essential cellular components such as membranes, proteins, and organelles. This process requires an increased intake of nutrients, particularly glucose, which not only fuels ATP production but also provides metabolic intermediates essential for biosynthesis. Additionally, amino acids play a crucial role as building blocks for protein synthesis, supporting the structural and functional needs of dividing cells.

Importantly, proliferating cells can reroute carbons from glycolysis at the pyruvate or lactate stage into anabolic pathways, such as lipid and nucleotide synthesis, rather than fully oxidizing them in the mitochondria. This metabolic flexibility allows cells to balance energy production with the synthesis of macromolecules necessary for growth and division, reinforcing the role of aerobic glycolysis in supporting rapid proliferation [16,17].

To keep growing and proliferating, tumor cells must ingest an overabundance of glucose. GLUT1 is an energy-free glucose transporter. Although low levels of GLUT1 expression are found in normal tissues and benign lesions, high levels of expression are frequently associated with carcinogenesis and can suggest a bad prognosis or recurrence [16].

The current study was conducted to examine the expression of GLUT1 in oral epithelial dysplasia and various histological grades of OSCC to assess its efficacy as a prognostic marker.

In the current study, poorly differentiated OSCC cases had the highest mean area percentage (47.37), while well-differentiated OSCC cases had the lowest value (13.42).

The current work investigated the expression of GLUT1 in oral epithelial dysplasia. GLUT1 expression was observed in the basal and suprabasal layers of the epithelium, but it was reduced in the most superficial layers. GLUT1 expression was observed mainly in the periphery of the tumor. These findings aligned with the study by Ehtisham et al. [8] and Patlolla et al., in 2020 [14].

The elevated expression of GLUT1 in oral epithelial dysplasia than in well-differentiated OSCC in present research is similar to that found in the study of Pereira et al. [16]; this overexpression is observed in conditions when cells require glucose as an energy source, such as during ischemia or hypoxia. These conditions might arise due to inadequate oxygen supply caused by poor blood movement [18,19].

We examined the distribution of GLUT1 immunostaining across invading islands in OSCC grades. In WDOSCC, GLUT1 expression was most noticeable on the tumor islands’ periphery and nonexistent in central keratin pearls. These findings agree with Patlolla et al. [14], who explained that greater glycogen buildup in keratin pearls is inversely associated with GLUT1 expression, indicating that mature and differentiated cells in keratinized areas do not express GLUT1. Glycogen is related to squamous epithelium cellular maturation but vanishes during neoplastic transformation as differentiation occurs. An antistromal staining pattern in PDOSCC areas lacking squamous development and keratinization may be produced by hypoxia-driven GLUT1 activation.

In the current study, the intracellular localization of GLUT1 expression showed a major transition from being primarily located in the cell membrane to being located in the cytoplasm and eventually to a combination of both membrane and cytoplasmic positives. The results of this investigation were consistent with the research conducted by Airley et al. [20], which investigated the relationship between the presence of GLUT1 in tumors and the duration and severity of hypoxia in various regions. Their proposal suggests that the presence of GLUT1 close to the Golgi apparatus results in the simultaneous production of this protein in both the cell membrane and the cytoplasm.

Recent research targeting GLUT1 has demonstrated the potential to improve prognosis and increase survival rates. However, additional studies using larger sample sizes are necessary to determine the clinical relevance of GLUT1 as a prognostic marker for evaluating the risk and prognosis of OSCC [21].

GLUT-1 expression did not show a significant correlation with factors such as gender, age, alcohol consumption, or regional recurrence. However, it was prejudiced by proliferation rate, oxygen availability, malignant transformation, tumor progression, histological grading, clinical staging, lymph node involvement, tobacco use, and distant metastasis [22]. In their analysis, Yu M et al. reported that GLUT-1 overexpression was linked to increased aggressiveness and invasive potential of the lesion [23].

The use of ImageJ software for quantitative measurement of GLUT1 expression offers significant advantages over traditional semi-quantitative scoring methods. Unlike subjective visual assessment, which is prone to interobserver variability, ImageJ provides objective and reproducible quantification of immunohistochemical staining by analyzing pixel intensity and area percentage. This automated approach can reduce human bias and enhance evaluation accuracy in histological examination and immunohistochemical assessment [24].

Despite the strengths of this study on Egyptian patients, certain limitations should be acknowledged. First, the sample size was relatively small, which may limit the generalizability of our findings to a broader population. Larger, multi-center studies are needed to validate these results. Second, the study design was retrospective, which prevents the establishment of a definitive causal relationship between the observed variables. A longitudinal study would provide more robust evidence regarding the temporal sequence of these associations in addition to future studies incorporating functional validation through in vitro assays or exploring co-expression patterns with additional hypoxia-related markers to further elucidate GLUT1’s role in OSCC progression. Future studies could also explore whether GLUT1 expression varies significantly between different intraoral subsites to provide additional insights into tumor behavior and prognosis. Lastly, while we use immunohistochemistry to assess Glut1, this approach has inherent limitations, including potential variability in interpretation and technical constraints, complementary genetic testing could provide additional insights.

## 5. Conclusions

The level of GLUT1 expression was elevated from oral epithelial dysplasia to various histological grades of OSCC. The presence of GLUT1 in actively dividing cells could indicate the tumor’s aggressiveness and susceptibility to different treatment approaches. More expression of this marker indicates more division and proliferation under hypoxic conditions.

## Figures and Tables

**Table 1:** Difference in mean GLUT1 area between different groups using ANOVA statistical test.

Groups		Mean GLUT1 Area%				
	Mean	SD	Min	Max	F-Value	*p*-Value
**OED**	17.13	3.02	14.209	20.239	348.026	<0.0001 *
**WDSCC**	13.42	3.309	10	17.208		
**MDSCC**	38.78	3.692	35.887	42.939		
**PDSCC**	47.37	2.392	43.913	50.552		

* significant at *p* < 0.05.

## Data Availability

All data generated or analyzed in this study are included in this published article. The data are available on request.
